# Introducing helmet non-invasive ventilation during COVID-19 pandemic: Early experience of two centres

**DOI:** 10.3389/fmed.2023.1075797

**Published:** 2023-02-07

**Authors:** Dipayan Chaudhuri, Rishi Sharma, Karen E. A. Burns, Joshua Piticaru, Deborah J. Cook, Bram Rochwerg

**Affiliations:** ^1^Department of Medicine, McMaster University, Hamilton, ON, Canada; ^2^Department of Health Research Methods, Evidence and Impact, McMaster University, Hamilton, ON, Canada; ^3^Interdepartmental Division of Critical Care Medicine, University of Toronto, Toronto, ON, Canada; ^4^Li Ka Shing Knowledge Institute, Unity Health Toronto – St. Michael’s Hospital, Toronto, ON, Canada; ^5^Division of Critical Care, Department of Medicine, St. Joseph’s Hospital, Syracuse, NY, United States; ^6^Department of Critical Care Medicine, St. Joseph’s Hospital, Hamilton, ON, Canada

**Keywords:** non-invasive ventilation, respiratory insufficiency, helmet, observational studies, COVID-19

## Abstract

**Purpose:**

The helmet is a novel interface for delivering non-invasive ventilation (NIV). We conducted a case series to characterize introduction of the helmet interface in both COVID and non-COVID patients at two-centres.

**Methods:**

We enrolled all patients with respiratory failure admitted to the Juravinski Hospital (Hamilton, Canada) and St. Joseph’s Health Center (Syracuse, New York) between November 1, 2020 and June 30, 2021 who used the helmet interface (Intersurgical StarMed) as part of this introduction into clinical practice. We collected patient demographics, reason for respiratory failure, NIV settings, device-related complications and outcomes. We report respiratory therapist’s initial experiences with the helmet using descriptive results.

**Results:**

We included 16 patients with a mean age of 64.3 ± 10.9 years. The most common etiology for respiratory failure was pneumonia (81.3%). The median duration of NIV during the ICU admission was 67.5 (15.3, 80.8) hours, with a mean maximum PS of 13.9 ± 6.6 cm H2O and a mean maximum PEEP of 10.4 ± 5.1 cm H20. Three patients (18.7%) did not tolerate the helmet. Ten (62.5%) patients ultimately required intubation, and 7 (43.4%) patients died while in the ICU. The most common reason for intubation was worsening hypoxia (70%). No adverse events related to the helmet were recorded.

**Conclusion:**

Over the 8-month period of this study, we found that the helmet was well tolerated in over 80% of patients, although, more than half ultimately required intubation. Randomized controlled trials with this device are required to fully assess the efficacy of this interface.

## Introduction

Non-invasive positive pressure ventilation (NIV) has been increasingly used as an alternative to endotracheal intubation and subsequent invasive mechanical ventilation (1), especially for those with less severe respiratory disease. NIV has been shown to reduce morbidity and mortality when compared to standard oxygen therapy, and in some cases, invasive mechanical ventilation (1). This is especially true in patients with chronic obstructive pulmonary disease (COPD), pulmonary edema and more recently in acute hypoxic respiratory failure due to COVID-19 (2). The most recent ERS/ATS guideline strongly recommends NIV for patients who have acute respiratory failure due to chronic obstructive pulmonary disease (COPD) exacerbations or cardiogenic pulmonary edema, and conditionally recommends NIV in patients with acute respiratory failure of a variety of other causes including trauma, post-operative respiratory failure and in patients who are immunocompromised (3).

Typically, NIV is delivered through a face mask interface; however, at higher pressures, the face mask can be difficult to tolerate and can cause significant air leak, thus impairing oxygenation and ventilation (4). Furthermore, patients using the face mask interface often feel claustrophobic and delirious patients may have difficulty keeping the mask in place; communication and nutrition therapy can also be challenging (5). The helmet is a relatively new interface used to deliver NIV in patients with respiratory failure. A transparent hood is placed over the entire head of the patient with a seal at the neck using a soft collar. The helmet reduces air leak and improves tolerability due to lack of contact with the patient’s face and better seal integrity at the neck (6). The ability to provide a better seal without obscuring the face also offers other potential advantages including enhanced comfort, and enabling oral intake, and permitting communication. In studies conducted using a breathing patient stimulator, the helmet was found to be safer in reducing respiratory virus dispersion, compared to facemask NIV or high flow nasal cannula (HFNC), making it particularly effective during pandemic-related illnesses, such as COVID-19, and severe acute respiratory syndrome (SARS) (7, 8).

Small trials in selected populations (ARDS, COVID-19 respiratory failure, community acquired pneumonia) have shown that the helmet is better tolerated, and associated with lower intubation rates, lower mortality, more ventilator free days and shorter ICU length of stay (4, 9–11). A recent network meta-analysis of randomized control trials (RCTs) comparing all non-invasive oxygenations strategies in 3804 patients with acute hypoxemic respiratory failure showed lower mortality with helmet NIV when compared to conventional oxygen therapy although this was based on low certainty evidence (12). We recently performed a systematic review and meta-analysis comparing the helmet and facemask interface for NIV that found the helmet may reduce mortality and intubation in both hypoxic and hypercarbic respiratory failure, although these conclusions were based on low certainty evidence (13).

While the helmet interface has been increasingly used in Europe, especially during the COVID pandemic (14), a lack of large-scale, well designed and adequately powered studies to inform practice on potential risks and benefits has limited its wider adoption. With regulatory approval now across North America, it is possible that helmet NIV could play an important role in the management of acute respiratory failure. Given this, interested ICUs have begun gaining experience with this technology to build familiarity and work towards a pilot randomized clinical trial (RCT) addressing the optimal interface in critically ill patients requiring NIV. The objective of this study was to describe our initial experience using the helmet interface for NIV among patients with acute respiratory failure in 2 tertiary care ICUs. Herein, we report patient characteristics, reasons for respiratory failure, NIV initiation and settings, device-related complications and patient outcomes.

## Materials and methods

### Study design

We enrolled all patients with acute respiratory failure admitted to the Juravinski Hospital ICU in Hamilton, Ontario, Canada and St Joseph’s Health Center in Syracuse, New York who were cared for using the helmet interface (Intersurgical StarMed) for delivery of NIV.

We obtained research ethics board approval using a waived consent model and retrospective data collection at both sites (Hamilton Integrated Research Ethics Board # 2021-13, 066-C, St. Joseph’s Health Center Integrate Research Ethics Board # 20-1,221-1). We provided training and educational material for helmet initiation and setup to both study sites prior to rollout. Primarily, this was done through multiple orientation sessions with a device representative from Intersurgical Canada and respiratory therapists, physicians and other healthcare professionals who were interested in learning about the new device. One free sample of the helmet device was donated to each centre and the helmet was first tried briefly on a healthy volunteer (in this case, the study author) to build familiarity with the operation of the device. Subsequently, the helmet was then used on actual patients with acute respiratory failure. Further details on exactly how the helmet was set up and used is included in the Supplementary materials.

We report findings using the STROBE checklist (Supplementary materials) (15). We included patients who were 18 years of age or older and were admitted to a critical care unit with either hypoxemic or hypercarbic respiratory failure of any etiology and were treated with NIV through the helmet interface. Patients were admitted between November 1, 2020 and June 30, 2021, which represents the period between when the helmet was first introduced at both sites and the initiation of a pilot RCT comparing helmet versus facemask interface for NIV delivery in patients with acute respiratory failure at one site (Juravinski Hospital) during the height of the COVID-19 pandemic in Ontario.

### Data collection

We developed and pilot tested a standardized case report form (CRF) for data collection. DC and RS collected data from the medical record in duplicate using medical record numbers (MRNs). Discrepancies were resolved through discussion and/or adjudicated by a third author if necessary. We collected baseline demographic data for all study patients including age, sex, BMI, APACHE II score, comorbidities, reasons for respiratory failure (patients could have more than one reason for respiratory failure) and initial respiratory parameters. For NIV, we collected duration of therapy, the number of episodes of therapy each patient received, the initial NIV settings and the maximum (highest) NIV settings for each patient. We collected positive end-expiratory pressure (PEEP), pressure support above PEEP (PS), fraction of inspired oxygen (FiO2) and tidal volume. We also captured helmet size, and co-interventions such as other non-invasive oxygen support and inotropes/vasopressors. In terms of patient variables, we collected respiratory parameters such as respiratory rate (RR), percentage saturation of O_2_ (S_P_O_2_), PaO_2_ (mmHg), and PaCO_2_ (mmHg) at initiation, 30 min after helmet NIV initiation and just before the helmet was discontinued or the patient was intubated. If the helmet was used multiple times in a single patient, we recorded each segment of use as an individual episode. We captured episodes in which the helmet was removed including reasons for removal (e.g., due to intolerance) and any technical problems with the usage of the helmet noted by the physician or respiratory therapist.

We collected patient outcomes including need for endotracheal intubation (time of intubation and reason for intubation in those requiring intubation), ICU and hospital mortality, ICU and hospital length of stay and adverse events related to helmet therapy. Serious adverse events with the helmet including bradycardia, hypotension, cardiac arrest, vomiting or aspiration were recorded by reviewing respiratory therapist (RT) notes in the patient’s medical record during helmet application. Finally, we solicited informal, in-person comments from 5 RTs, including the RT lead, at one site regarding their initial impressions of the helmet device.

### Statistical analysis

We performed descriptive analysis of the patients included in this case series. We summarized data using means with standard deviations, medians with interquartile ranges as appropriate, and counts with percentages. All statistical analysis were performed with Microsoft Excel, version 16.4.3.

## Results

### Baseline demographics

We included 16 eligible patients (13 from the Hamilton site and 3 from the Syracuse site). Of these, 5 (34.4%) were female and the mean (standard deviation) age was 64.3 ± 10.9 years (Table 1). The mean APACHE II score was 9.7 ± 4.9. Patients were placed on helmet NIV an average of 68.6 ± 56.7 h from the time of hospital admission. The most common patient comorbidities included smoking (37.5%), COPD (31.3%), cancer (31.3%) and diabetes mellitus type 2 (31.3%) (Table 1). The most common causes of respiratory failure were pneumonia (81.3%), COVID-19 (62.5%) and ARDS (50%) (Table 1). One patient was on vasopressors when helmet NIV was initiated.

**Table 1:** Baseline demographics of included patients.

Characteristic	All patients
**Demographic**	
Average age	64.3 ± 10.9
# of male (%)	11 (68.8)
# of female (%)	5 (31.3)
Average APACHE II score	9.7 ± 4.9
Time to NIV from admission (hours)	68.6 ± 56.7
**Medical history**	
Cardiovascular disease (%)	3 (18.8)
Asthma (%)	2 (12.5)
COPD (%)	5 (31.3)
T2DM (%)	5 (31.3)
Smoker (%)	6 (37.5)
Cancer (%)	5 (31.3)
Immunocompromised (%)	2 (12.5)
**Etiology of respiratory failure**	
Pneumonia (%)	13 (81.3)
ARDS (%)	8 (50.0)
Pulmonary edema (%)	1 (6.3)
Aspiration (%)	1 (6.3)
Postoperative (%)	0 (0)
Pulmonary embolism (%)	0 (0)
Toxic (%)	1 (6.3)
Neuromuscular disease (%)	0 (0)
AECOPD (%)	2 (12.5)
COVID-19 (%)	10 (62.5)

Mean (standard deviation) for continuous variables. NIV, non-invasive ventilation; COPD, chronic obstructive pulmonary disease; T2DM, type 2 diabetes mellitus; ARDS, acute respiratory distress syndrome; AECOPD, COPD exacerbation.

### NIV initiation and settings

**Figure 1:**
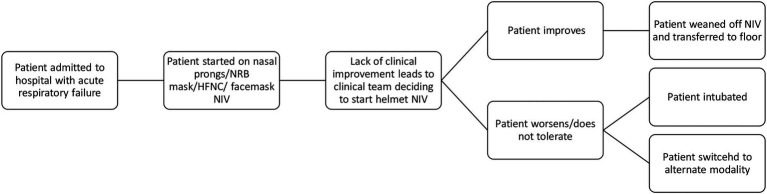
Clinical trajectory of a patient receiving helmet NIV. HFNC, high flow nasal cannula; NIV, non-invasive ventilation; NRB, non-rebreather.

Within a month of our initial training sessions, helmet utilization commenced at both sites. Figure 1 illustrates a patient’s possible clinical trajectory when the helmet was used. Eight (50%) of the included patients were initially treated with HFNC before being placed on helmet NIV, with the remainder being trialed on either nasal prongs (NP) (4 patients) or non-rebreather mask (NRB) (2 patients) prior to helmet initiation. Of note, one patient was extubated directly to helmet NIV, and one patient was initially started on facemask NIV before being switched to helmet NIV.

The median (IQR) duration of helmet NIV was 67.5 (15.3, 80.8) hours with the mean initial PEEP being set at 8.4 ± 2.4 cm H2O and the mean initial pressure support (PS) being set at 12.9 ± 4.1 cm H2O (Table 2). The mean maximum PEEP was 10.4 ± 5.1 cm H20 with the mean maximum PS being 13.9 ± 6.6 cm H2O. The initial mean FiO2 settings were 56.6% ± 23.4%. Heat and moisture exchanges (HME) were used to humidify the inhaled air for all patients. The trigger threshold for all patients were set to be as sensitive as possible without allowing for auto-triggering. Table 3 illustrates patient respiratory parameters before and after starting helmet NIV.

**Table 2:** Average NIV parameters.

Average duration (hours) (mean ± SD)	74.1 ± 81.9
(median, IQR)	67.5 (15.3, 80.8)
Average initial PEEP (cm H20)	8.4 ± 2.4
Average initial P_Support_ (cm H20)	12.9 ± 4.1
Average maximum PEEP (cm H20)	10.4 ± 5.1
Average maximum P_Support_ (cm H20)	13.9 ± 6.6
Average respiratory rate (mean ± SD)	26.1 ± 5.2

Results in mean (standard deviation), unless otherwise stated. IQR, interquartile range; cm H20, centimetres of water; PEEP, positive end expiratory pressure; P_support_, pressure support.

**Table 3:** Respiratory parameters before and after helmet initiation.

	Before helmet NIV initiation		After helmet NIV initiation	
	Mean (SD)	Median (IQR)	Mean (SD)	Median (IQR)
Oxygen saturation (SpO2)	93.8 ± 3.3	93.0 (92.0, 94.3)	93.1 ± 3.3	93.0 (91.0, 94.0)
Fraction of inspired oxygen (FiO2)	67.9 ± 28.5	67.5 (36.0, 96.3)	58.4 ± 22.1	50.0 (40.0, 75.0)
Partial venous pressure of carbon dioxide (mm Hg) (PvCO2)	54.9 ± 29.2	43.0 (37.5, 62.8)	60.5 ± 30.2	49.0 (41.0, 68.0)
Respiratory rate (breaths/min)	28.4 ± 9.2	28.0 (21.0, 36.3)	26.2 ± 5.2	26.0 (22.0, 28.0)

cm H20, centimetres of water.

### Outcomes

Table 4 outlines patient outcomes. Of those treated with helmet, 10/16 (62.5%) patients were ultimately intubated. The primary reason for intubation was hypoxia (70%). In those that required intubation, patients received helmet NIV for a median (IQR) of 42.0 (5.5, 72.0) hours before intubation. Three (18.7%) patients did not tolerate helmet NIV due to increased agitation and were switched to facemask NIV (*n* = 2) or HFNC (*n* = 1). All 3 of these patients had COVID ARDS and were subsequently intubated after failing this alternative non-invasive oxygen support. Of those enrolled, 7/16 (43.4%) patients died in ICU, and 9/16 (56.3%) died in hospital. The mean duration of stay for all patients in the ICU was 30.1 ± 28.4 days and the mean duration of hospital stay was 36.1 ± 29.9 days. There were no serious adverse events reported with the helmet.

**Table 4:** Patient outcomes.

# of Intubations (%)	10 (62.5)
# ICU mortality (%)	7 (43.4)
# Hospital mortality (%)	9 (56.3)
Average LOS in ICU (days)	30.1 ± 28.4
Average LOS in hospital (days)	36.1 ± 29.9
**Etiology of intubation**	
Hypoxia (%)	7 (43.4)
Neurologic failure (%)	1 (6.3)
Respiratory failure (%)	1 (6.3)
Circulatory failure (%)	1 (6.3)

Results in mean (standard deviation) for continuous variables. ICU, intensive care unit; LOS, length of stay.

All individual patient demographics, NIV settings and outcomes are outlined in the Appendix.

### RT impressions

Common concerns amongst the RTs regarding helmet use as recorded from their informal comments were primarily regarding oral dryness, along with skin breakdown and discomfort at the armpits.

## Discussion

We found that helmet NIV was generally well tolerated by patients and adopted by healthcare staff. While over half of the patients treated with helmet NIV were ultimately intubated, primarily because of hypoxia, there were no increase in adverse events recorded with helmet use. Overall, the helmet appears to be a viable and feasible alternative to facemask NIV, although large randomized trials examining this interface are necessary to better elucidate the benefits, risk, and comfort in critically ill patients with acute respiratory failure.

Most included patients were able to tolerate long periods of continuous NIV using the helmet interface without an increase in adverse events. While three patients were unable to tolerate the helmet and were switched to alternative respiratory support devices, it is important to note that all three of these patients were subsequently intubated. Thus, it is uncertain whether the patients did not tolerate the helmet itself, or whether they would have required intubation regardless of modality of non-invasive respiratory support. Commonly described complications of NIV, including ventilation associated pneumonia (VAP), and gastric distension or emesis (13), were not observed within this series, although this may have been related to careful selection of patients or a reflection of the small numbers enrolled.

Despite the increased dead space associated with the helmet, ventilator settings were well within the normal range for NIV. It is important to note that the helmet device did not allow for the monitoring of patient respiratory mechanics such as inspiratory effort, tidal volume or overall mechanical power. Thus, it could not be determined whether patients were developing patient-self inflicted lung injury (P-SILI) during long periods of helmet NIV.

One of the included patients in the study was started on helmet NIV prophylactically (without established respiratory failure) following extubation. While this case was different from the others, we felt it important to include this data as NIV has shown some benefit when used as prophylaxis in patients at high risk of extubation failure (3). This specific patient ended up requiring re-intubation due to circulatory failure and worsening hypercarbia.

One of the most common concerns regarding the helmet interface, as compared to facemask, is increased risk of hypercapnia and CO2 recirculation (16, 17). While previous studies have shown that patients with severe pneumonia and COPD have a better response to NIV (18), in this case series, which included five patients with a history of COPD, we demonstrated a small median increase in PCO2 (from 43 mm Hg to 49 mm Hg) following initiation of patients on helmet NIV. The reliability of this finding is questionable given the small numbers. Also, two of the patients who failed helmet NIV (patients number 4 and 13 in the Supplementary Appendix) had a subsequent large rise in their CO2 following helmet initiation, disproportionally increasing the median post-therapy CO2 level across all patients. Of the two patients who were specifically started on helmet NIV for COPD exacerbations and hypercapnia, one experienced an improvement in CO2 levels with helmet NIV whereas the other experienced an increase in CO2 levels and ultimately died. While the small sample size and possibility of selection bias prevents us from making any definitive conclusions regarding the effectiveness of helmet NIV in patients with COPD exacerbations or hypercarbia, the data supports the need for further study of this population in larger prospective trials or randomized controlled studies.

More than half of the patients included in this case series (62.5%) were eventually intubated and 56.3% of them died. Recent observational studies and RCTs enrolling a similar patient population have shown lower rates of intubation and mortality (19–21). Moreover, a systematic review and meta-analysis of randomized controlled trials that compared the outcomes of patients treated with helmet NIV to facemask NIV demonstrated lower rates of intubation and mortality as compared to this case series (13). Therefore, whether the findings from this case series, which describes a population with a high severity of illness, are generalizable to a less hypoxemic population remains uncertain. We believe there are a few explanations for the high illness severity in this study population. First, in both study centers, patients with hypoxic respiratory failure are typically initiated on HFNC if standard oxygen therapy fails, as was seen in over 50% of those enrolled. Therefore, patients initiated on helmet NIV had already failed one type of non-invasive oxygen support (e.g., HFNC), suggesting they were sicker than the populations examined in most other primary NIV studies. Second, a majority of study patients had respiratory failure due to COVID-19 and were enrolled during the height of the COVID-19 pandemic. Patients with respiratory failure due to COVID-19 may have worse outcomes as compared to those without COVID-19 (22) and lack of resources, along with increased healthcare burden may have also contributed to poor outcomes in this population.

Strengths of this study include a pragmatic design where clinicians were encouraged to apply the helmet in patients who they considered clinically appropriate and to titrate settings as indicated, with a protocol being available only as guidance. Since the purpose of this study was to describe our experience with this new technology and also build competence and comfort with it, this approach ensured that both objectives were met. While previous observational studies and RCTs examining the helmet included patients with specific aetiologies of respiratory failure, limiting the external validity, this study included patients with various aetiologies of acute respiratory failure. Moreover, while informally obtained, we are the first to collect and report respiratory therapist feedback on helmet use and operationalization. Finally, despite the challenges of incorporating a new respiratory support technology in a healthcare setting during a viral respiratory pandemic, we included a large percentage of COVID-19 related hypoxic respiratory failure (62.5% of patients). This study also has important limitations. First, given that this was a case series, no inferences regarding causation or efficacy or harm are possible due to confounding. Given that clinicians chose which patients were cared for using the helmet interface, selection bias remains an important possibility. The case series includes a relatively small number of patients and we did not measure or report respiratory mechanics. Further, we were unable to capture all relevant co-interventions, particularly, with respect to medications. Finally, while we sought informal feedback respiratory therapists, we did not conduct structured interviews with RTs or solicit the impressions of physicians, nurses or patients.

## Conclusion

Over the 8-month period of this study, we found that the helmet was well tolerated in over 80% of patients, although, more than half ultimately required intubation. Randomized controlled trials with this device are required to fully assess the efficacy of this interface.

## Data availability statement

The original contributions presented in the study are included in the article/supplementary material, further inquiries can be directed to the corresponding author/s.

## Ethics statement

The studies involving human participants were reviewed and approved by Hamilton Integrated Research Ethics Board # 2021-13066-C, St. Joseph’s Health Center Integrate Research Ethics Board # 20-1221-1. Written informed consent for participation was not required for this study in accordance with the national legislation and the institutional requirements.

## Author contributions

DCh and BR initially conceived of this study. DCh and BR wrote up the study protocol and submitted for ethics approval. DCh and RS performed data collection at Juravinski Site. JP performed data collection at the St. Joseph site. DCh and RS did the statistical analysis. DCh, DCo, KB, and BR wrote up the manuscript. All authors contributed to the article and approved the submitted version.

## Conflict of interest

The authors declare that the research was conducted in the absence of any commercial or financial relationships that could be construed as a potential conflict of interest.

## Publisher’s note

All claims expressed in this article are solely those of the authors and do not necessarily represent those of their affiliated organizations, or those of the publisher, the editors and the reviewers. Any product that may be evaluated in this article, or claim that may be made by its manufacturer, is not guaranteed or endorsed by the publisher.
