# Supplementary material for: Introducing helmet non-invasive ventilation during COVID-19 pandemic: Early experience of two centres
Source: Front Med (Lausanne). 2023 Feb 7;10:1075797. doi: 10.3389/fmed.2023.1075797 (PMC9941518; doi:10.3389/fmed.2023.1075797)
Supplement: Supplementary file 1 [file Data_Sheet_1.docx]

STROBE Statement—Checklist of items that should be included in reports of ***cohort studies***

|  | Item No | Recommendation | Page No |
| --- | --- | --- | --- |
| **Title and abstract** | 1 | (*a*) Indicate the study’s design with a commonly used term in the title or the abstract | 1,2 |
|  |  | (*b*) Provide in the abstract an informative and balanced summary of what was done and what was found |  |
| Introduction | | | |
| Background/rationale | 2 | Explain the scientific background and rationale for the investigation being reported | 4 |
| Objectives | 3 | State specific objectives, including any prespecified hypotheses | 5 |
| Methods | | | |
| Study design | 4 | Present key elements of study design early in the paper | 6 |
| Setting | 5 | Describe the setting, locations, and relevant dates, including periods of recruitment, exposure, follow-up, and data collection | 6 |
| Participants | 6 | (*a*) Give the eligibility criteria, and the sources and methods of selection of participants. Describe methods of follow-up | 6 |
|  |  | (*b*) For matched studies, give matching criteria and number of exposed and unexposed |  |
| Variables | 7 | Clearly define all outcomes, exposures, predictors, potential confounders, and effect modifiers. Give diagnostic criteria, if applicable | 6,7 |
| Data sources/ measurement | 8* | For each variable of interest, give sources of data and details of methods of assessment (measurement). Describe comparability of assessment methods if there is more than one group | 6,7 |
| Bias | 9 | Describe any efforts to address potential sources of bias | N/A |
| Study size | 10 | Explain how the study size was arrived at | N/A |
| Quantitative variables | 11 | Explain how quantitative variables were handled in the analyses. If applicable, describe which groupings were chosen and why | 7 |
| Statistical methods | 12 | (*a*) Describe all statistical methods, including those used to control for confounding | 7 |
|  |  | (*b*) Describe any methods used to examine subgroups and interactions |  |
|  |  | (*c*) Explain how missing data were addressed |  |
|  |  | (*d*) If applicable, explain how loss to follow-up was addressed |  |
|  |  | (*e*) Describe any sensitivity analyses |  |
| Results | | |  |
| Participants | 13* | (a) Report numbers of individuals at each stage of study—eg numbers potentially eligible, examined for eligibility, confirmed eligible, included in the study, completing follow-up, and analysed | 8 |
|  |  | (b) Give reasons for non-participation at each stage |  |
|  |  | (c) Consider use of a flow diagram |  |
| Descriptive data | 14* | (a) Give characteristics of study participants (eg demographic, clinical, social) and information on exposures and potential confounders | 8 |
|  |  | (b) Indicate number of participants with missing data for each variable of interest |  |
|  |  | (c) Summarise follow-up time (eg, average and total amount) |  |
| Outcome data | 15* | Report numbers of outcome events or summary measures over time | 9 |

| Main results | 16 | (*a*) Give unadjusted estimates and, if applicable, confounder-adjusted estimates and their precision (eg, 95% confidence interval). Make clear which confounders were adjusted for and why they were included | 8-9 |
| --- | --- | --- | --- |
|  |  | (*b*) Report category boundaries when continuous variables were categorized |  |
|  |  | (*c*) If relevant, consider translating estimates of relative risk into absolute risk for a meaningful time period |  |
| Other analyses | 17 | Report other analyses done—eg analyses of subgroups and interactions, and sensitivity analyses | N/A |
| Discussion | | | |
| Key results | 18 | Summarise key results with reference to study objectives | 10 |
| Limitations | 19 | Discuss limitations of the study, taking into account sources of potential bias or imprecision. Discuss both direction and magnitude of any potential bias | 11 |
| Interpretation | 20 | Give a cautious overall interpretation of results considering objectives, limitations, multiplicity of analyses, results from similar studies, and other relevant evidence | 11 |
| Generalisability | 21 | Discuss the generalisability (external validity) of the study results | 11 |
| Other information | | | |
| Funding | 22 | Give the source of funding and the role of the funders for the present study and, if applicable, for the original study on which the present article is based | N/A |

*Give information separately for exposed and unexposed groups.

**Note:** An Explanation and Elaboration article discusses each checklist item and gives methodological background and published examples of transparent reporting. The STROBE checklist is best used in conjunction with this article (freely available on the Web sites of PLoS Medicine at http://www.plosmedicine.org/, Annals of Internal Medicine at http://www.annals.org/, and Epidemiology at http://www.epidem.com/). Information on the STROBE Initiative is available at http://www.strobe-statement.org.

**StarMed NIV Helmet Usage**

- Single patient use (for up to 7 days)
- Must be used with **dual limb** vent circuit
  - **Servo-i**
    - Connect inspiratory limb to one port and expiratory limb to opposite port
      - Ports are interchangeable (doesn’t matter which limb goes to which port)
  - **Hamilton G5**
    - Flow sensor, patient wye, and dual limb circuit to one port & cap off opposite port
      - Use blue cap from heated circuit to block opposite port
  - **Do not** use StarMed helmet with the **V60**
- Only use helmet with a **dry circuit** – heated humidity will cause excessive buildup of condensation
- Smooth bore tubing recommended for noise reduction (but can use standard circuits vent circuits)
- Place a special **low resistance filter** at the inspiratory port of the helmet for noise reduction
  - A standard filter will create too much resistance
- Determine appropriate helmet size by using supplied measuring tape around patient’s neck to measure neck circumference
- Requires 2 people to place helmet on patient’s head
  - Open large access port & check one-way valve
  - Pull back film/seal on either side when placing over head
  - Secure straps snuggly under patient’s arms
    - Adjust length of straps so that rigid ring is about 1 cm from patient’s shoulders
  - Use bulb from art line pressure bag to inflate inner neck cushion for comfort
  - Close large access port & start vent to inflate/pressurize helmet
- **NIV mode** is recommended
  - Consider **invasive PSV** to access/adjust additional parameters if needed (ie: trigger, ramp, etc)
- Must set **minimum parameters** to ensure **CO2 clearance**:
  - **PEEP 5 cmH20 (minimum)**
  - **PS 12 cmH2O (minimum)**
  - Some pressure will be lost to the helmet – may need to set parameters higher than expected
    - Consider setting PEEP & PS **30%-50% higher** than you usually would
- titrate PS to RR <25 bpm + decreased accessory muscle use and PEEP to sats >90% (and >88% for COPD)
- Set **trigger** as **sensitive** as possible (without inducing auto triggering)
- Set **ramp/slope** as **fast** as possible (titrate to patient comfort)
- Displayed **volumes** will be inaccurate
  - Vt will be much larger than normal because helmet is considered the “lung”
    - 50%-75% of the Vt delivered is distributed to the helmet
  - You can trend the Vt but do not assume this is what the patient’s lungs are receiving
- Adjust alarm limits appropriately
- ETCO2 monitoring
  - Cuvette can be placed at expiratory outlet of the helmet
  - Consider measuring ETCO2 inside the helmet using ETCO2 nasal cannula

Supplementary Table 1: Baseline Characteristics and Reason for NIV

|  | **Demographics** | | | **Past Medical History** | | | | | | | **Cause of Respiratory Failure** | | | | | | | | | |
| --- | --- | --- | --- | --- | --- | --- | --- | --- | --- | --- | --- | --- | --- | --- | --- | --- | --- | --- | --- | --- |
| **Study ID number** | **Age** | **Gender** | **APACHE II** | **Cardiovascular disease (Y/N)** | **Asthma (Y/N)** | **COPD (Y/N)** | **DM (Y/N)** | **Smoker (Y/N)** | **Cancer (Y/N)** | **Immunocompromised (Y/N)** | **Pneumonia (Y/N)** | **ARDS (Y/N)** | **Pulmonary edema (Y/N)** | **Aspiration (Y/N)** | **Postoperative (Y/N)** | **Pulmonary embolism (Y/N)** | **Toxic (Y/N)** | **Neuromuscular disease (Y/N)** | **COPD (Y/N)** | **COVID (Y/N)** |
| 1 | 63 | F | 9 | N | Y | N | Y | N | N | N | Y | N | N | N | N | N | N | N | N | Y |
| 2 | 79 | M | 8 | N | N | N | N | N | N | Y | Y | N | N | Y | N | N | Y | N | N | N |
| 3 | 75 | M | 13 | N | N | Y | Y | Y | Y | N | Y | N | N | N | N | N | N | N | N | N |
| 4 | 85 | F | 16 | N | N | Y | N | Y | Y | N | N | N | N | N | N | N | N | N | Y | N |
| 5 | 55 | F | 3 | N | N | N | N | N | N | N | N | N | N | N | N | N | N | N | N | N |
| 6 | 67 | F | 7 | N | N | Y | Y | Y | Y | N | N | N | N | N | N | N | N | N | Y | N |
| 7 | 73 | M | 6 | N | N | N | N | N | N | N | Y | N | N | N | N | N | N | N | N | N |
| 8 | 77 | M | 19 | Y | N | Y | N | Y | N | N | Y | N | Y | N | N | N | N | N | N | Y |
| 9 | 56 | F | 13 | N | N | N | N | N | N | N | Y | Y | N | N | N | N | N | N | N | Y |
| 10 | 58 | M | 8 | N | N | N | Y | N | N | N | Y | Y | N | N | N | N | N | N | N | Y |
| 11 | 60 | M | 4 | N | N | N | N | N | N | N | Y | Y | N | N | N | N | N | N | N | Y |
| 12 | 56 | M | 4 | N | N | N | Y | N | N | N | Y | Y | N | N | N | N | N | N | N | Y |
| 13 | 58 | M | 5 | Y | N | N | N | Y | Y | Y | Y | Y | N | N | N | N | N | N | N | Y |
| 14 | 54 | M | 11 | N | N | N | N | Y | N | N | Y | Y | N | N | N | N | N | N | N | Y |
| 15 | 45 | M | 12 | N | Y | N | N | N | N | N | Y | Y | N | N | N | N | N | N | N | Y |
| 16 | 67 | M | 17 | Y | N | Y | N | N | Y | N | Y | Y | N | N | N | N | N | N | N | Y |

COPD – chronic obstructive pulmonary disease, DM – diabetes mellitus, ARDS – acute respiratory distress syndrome

Supplementary Table 2: Respiratory parameters

|  |  | **Respiratory parameters before putting helmet on** | | | | | | **Respiratory parameters 30 minutes after putting helmet on** | | | | | **Respiratory parameters before removing helmet** | | | | | | | |
| --- | --- | --- | --- | --- | --- | --- | --- | --- | --- | --- | --- | --- | --- | --- | --- | --- | --- | --- | --- | --- |
| **Study ID number** | **Total Duration (hours)** | **Initial therapy (HFNC/FM/NP,Venturi mask)** | **SPO2 (%)** | **FiO2 (%)** | **Inotropes/vasopressors (Y/N)** | **PvCO2 (mm Hg)** | **RR** | **RR** | **SPO2 (%)** | **FiO2 (%)** | **Ionotrope/vasopressor (Y/N)** | **PvCO2 (mm Hg)** | **RR** | **SPO2 (%)** | **FiO2 (%)** | **Ionotrope/vasopressor (Y/N)** | **PvCO2 (mm Hg)** | **Cessation due to Intolerance (Y/N)** | **Technical issues** | **Device transitioned to if intolerance** |
| 1 | 4 | HFNC | 94 | 95 | N | 38 | 26 | 28 | 89 | 75 | N | 38 | 38 | 91 | 95 | N | 38 | Y | N | HFNC |
| 2 | 72 | NP | 94 | 36 | Y | 61 | 18 | 40 | 99 | 30 | Y | 40 | 34 | 98 | 100 | Y | 61 | N | N | N/A |
| 3 | 73 | NP | 100 | 36 | N | 134 | 30 | 22 | 91 | 30 | N | 78 | 35 | 97 | 100 | N | 134 | N | N | N/A |
| 4 | 296 | BiPAP | 93 | 30 | N | 103 | 18 | 20 | 99 | 75 | N | 150 | 21 | 96 | 70 | N | 143 | N | N | N/A |
| 5 | 10 | HFNC | 93 | 55 | N | 55 | 15 | 22 | 96 | 80 | N | 61 | 19 | 96 | 70 | N | 50 | N | N | N/A |
| 6 | 148 | HFNC | 95 | 35 | N | 80 | 24 | 20 | 93 | 40 | N | 63 | 24 | 95 | 35 | N | 60 | N | N | N/A |
| 7 | 17 | HFNC | 93 | 94 | N | 36 | 41 | 26 | 92 | 45 | N | 37 | 21 | 93 | 45 | N | 36 | N | N | N/A |
| 8 | 75 | NRB | 98 | 100 | N | 68 | 40 | 26 | 93 | 45 | N | 68 | 40 | 98 | 100 | N | 68 | Y | N | Conventional BiPAP |
| 9 | 22 | NP | 91 | 80 | N | 41 | 31 | 24 | 88 | 90 | N | 49 | 31 | 91 | 100 | N | 41 | N | N | N/A |
| 10 | 3 | HFNC | 91 | 90 | N | 44 | 28 | 28 | 91 | 90 | N | 44 | 35 | 91 | 90 | N | 44 | Y | N | Conventional BiPAP |
| 11 | 206 | HFNC | 92 | 50 | N | 38 | 28 | 28 | 94 | 50 | N | 41 | 28 | 89 | 40 | N | 31 | N | N | N/A |
| 12 | 26 | NP | 94 | 36 | N | 24 | 16 | 28 | 93 | 70 | N | 46 | 22 | 92 | 40 | N | 50 | N | N | N/A |
| 13 | 63 | ETT | 100 | 50 | N | 50 | 22 | 28 | 92 | 40 | N | 71 | 24 | 94 | 92 | N | 129 | N | N | N/A |
| 14 | 72 | HFNC + NRB | 92 | 100 | N | 36 | 40 | NR | NR | NR | NR | NR | 33 | 93 | 100 | N | NR | N | N | N/A |
| 15 | 98 | HFNC + NRB | 87 | 100 | N | 42 | 42 | NR | NR | NR | NR | NR | 51 | 91 | 100 | N | NR | N | AIR LEAK AT CUFF DUE TO PATIENT'S BODY HABITUS | N/A |
| 16 | 1 | 15L NC + NRB | 93 | 100 | N | 29 | 35 | NR | NR | NR | NR | NR | 29 | 92 | 100 | N | NR | N | N | N/A |

HFNC – high flow nasal cannula, FM – facemask, NP – nasal prongs, SpO2 – oxygen saturation %, FiO2 – % of inspired O2, PvCO2 – venous carbon dioxide content in blood, RR – respiratory rate

Supplementary Table 3: Outcome data

| **Study ID number** | **Time from admission to helmet NIV initiation (hours)** | **Endotracheal intubation (Y/N)** | **If intubated, what was the reason** | **ICU Mortality (Y/N)** | **Hospital Mortality (Y/N)** | **ICU LOS (days)** | **Hospital LOS (days)** | **Adverse events** |
| --- | --- | --- | --- | --- | --- | --- | --- | --- |
| 1 | 113 | Y | Hypoxia | Y | Y | 100 | 108 | ARDS, bacteremia |
| 2 | 117 | Y | Hypoxia | Y | Y | 37 | 37 | S. epi bacteremia, aspiration |
| 3 | 5 | N |  | Y | Y | 4 | 4 | COVID-19 PNA |
| 4 | 4 | N |  | Y | Y | 12 | 12 | N/A |
| 5 | 144 | Y | Neurologic failure | N | N | 26 | 31 | N/A |
| 6 | 8 | N |  | N | N | 17 | 17 | S. epi bacteremia, R vulvar abscess |
| 7 | 29 | N |  | N | N | 7 | 16 | N/A |
| 8 | 2 | Y | Fatiguing/High RR | N | Y | 31 | 54 | delirium |
| 9 | 36 | Y | Hypoxia | N | N | 18 | 21 | B. cereus bacteremia, PE |
| 10 | 50 | Y | Hypoxia | Y | Y | 38 | 38 | N/A |
| 11 | 140 | N |  | N | N | 20 | 41 | N/A |
| 12 | 114 | N |  | N | N | 5 | 14 | N/A |
| 13 | 155 | Y | Circulatory failure | N | Y | 86 | 86 | N/A |
| 14 | 99 | Y | Hypoxia | Y | Y | 26 | 26 | NONE |
| 15 | 10 | Y | Hypoxia | N | N | 53 | 69 | NONE |
| 16 | 72 | Y | Hypoxia | Y | Y | 1 | 3 | NONE |

NIV – non-invasive ventilation, ICU – intensive care unit, LOS – length of stay
